# Seroprevalence of Cytomegalovirus among pregnant women and hospitalized children in Palestine

**DOI:** 10.1186/1471-2334-13-528

**Published:** 2013-11-09

**Authors:** Tahani Neirukh, Ayda Qaisi, Niveen Saleh, Areej Abu Rmaileh, Eman Abu Zahriyeh, Lina Qurei, Firas Dajani, Taghreed Nusseibeh, Hatem Khamash, Sabri Baraghithi, Maysa Azzeh

**Affiliations:** 1 Central Laboratory, Al-Makassed Islamic Charitable Hospital, East Jerusalem, Palestine; 2 MRC, Virology Research Laboratory, Al-Quds University, Abu Dies, East Jerusalem, Palestine; 3 Neonatal Department, Al-Makassed Islamic Charitable Hospital, East Jerusalem, Palestine; 4 Pediatric Department, School of Medicine, Al-Quds University, Abu Dies, East Jerusalem, Palestine

**Keywords:** Cytomegalovirus, HCMV seroprevalence, Pregnant women, Congenital HCMV infection

## Abstract

**Background:**

Human Cytomegalovirus (HCMV) is the most common cause of congenital infections. The maternal immune status plays a major role in the likelihood of congenital infection. The aim of this study is to shed light on the seroprevalence of HCMV in pregnant women, hospitalized children and newborns including cases of congenital infections in Palestine.

**Methods:**

We analyzed HCMV IgG and IgM test results that had been ordered for pregnant women, hospitalized children and newborns in the years 2006–2012 at Al-Makassed Islamic Charitable Hospital (MICH) in East Jerusalem. Furthermore, we reviewed the medical charts of newborns and HCMV IgM-positive children.

**Results:**

HCMV IgG was positive in 96.6% of pregnant women, in 88% of hospitalized children and in 98.4% of hospitalized newborns. HCMV IgM was positive in 11.5% of pregnant women, in 11.7% of hospitalized children and in 2% of hospitalized newborns respectively. The HCMV avidity assay revealed that 95% of IgM-positive pregnant women had high avidity (>60%) indicating that most Palestinian women were undergoing a recurrent HCMV infection. Real time PCR on limited number of cases indicated that 62.5% of infants, mostly born to IgM-positive mothers and 83.3% of HCMV IgM-positive children had detectable HCMV DNA in their urine. Out of the 249 newborns tested during this study period, four (1.6%) were subjected to Gancyclovir treatment because of symptomatic congenital HCMV infection.

**Conclusions:**

This is the first report to provide an insight into HCMV seroprevalence in Palestine. Despite the high rate of seropositivity, the importance of HCMV testing during pregnancy should not be underestimated. A comprehensive study with a long term follow-up examination of offspring born to HCMV IgM-positive mothers would be required to provide estimates of an accurate percentage of symptomatic congenital HCMV infection in Palestine.

## Background

Some of the most common infections associated with congenital anomalies are summarized in TORCH testing. TORCH includes Toxoplasmosis, Other (syphilis, varicella-zoster, parvovirus B19, Hepatitis B), Rubella, Cytomegalovirus (CMV), and Herpesvirus infections. Most of the TORCH infections cause mild maternal morbidity. However, they may have very serious consequences in the fetus, with a high rate of congenital anomalies, mental retardation, and an increased incidence of stillbirths [1,2].

Human cytomegalovirus (HCMV), HHV-5, belongs to the beta herpes family and is one of the most common causes of congenital viral infections. Congenital HCMV infection is associated with permanent hearing loss, vision loss and neurological impairment [3-5]. Maternal sexual behavior and contact with infected young children are the common source of infection [6]. Vertical transmission of HCMV to the fetus can be attributed to either recurrent maternal infection [7,8], or primary maternal infection [9]. Recurrent infection is the result of two possibilities: a reactivation of latent virus acquired prior to pregnancy or a reinfection with a new HCMV strain during pregnancy [10]. Compared with recurrent infection, primary HCMV infection accounts for the highest transplacental transmission rate to the fetus, causing a high likelihood of fetal damage [9,11-13]. Furthermore, the gestational age at which primary HCMV infection occurs is crucial; the earlier the infection occurs during pregnancy, the more severe the consequences [9,14,15].

The seroprevalence of HCMV is generally high in developing countries, and among those of lower socioeconomic status in developed countries [16]. HCMV seroprevalence status among pregnant and childbearing age women is the main focus of various worldwide studies due to the severe consequences to offspring. A relatively low seroprevalence, 40%-60%, is reported from Australia, Belgium, France, Germany and USA [17-23]. A high HCMV seroprevalence (>90%) is reported from Brazil, Taiwan [24-27] and in regional countries including Turkey, Qatar and Saudi Arabia [28-31]. Reports from USA and Israel indicate that the HCMV seroprevalence among women varies based on ethnical and/or racial groups [32-34].

In this study, we analyzed the results of HCMV-specific IgG and IgM assays at Al-Makassed Islamic Charitable Hospital (MICH) in East Jerusalem, the referral hospital in Palestine, and from the limited number of HCMV viral load records maintained at the Virology Research Laboratory, Medical Research Center, Al-Quds University, East Jerusalem, Palestine. The data presented here shed light on the incidence of HCMV infection in pregnant Palestinian women, children and newborns.

## Methods

### Study population

MICH is the referral hospital for Palestine and the main teaching hospital associated with Al-Quds University Medical School. Accordingly, patients visiting the hospital come from all over the West Bank, East Jerusalem and the Gaza strip. However, most pregnant women seeking consultation at MICH are predominantly residents of East Jerusalem district. The range of monthly deliveries at MICH is 180–200, most of which are not booked, i.e. they did not consult the MICH outpatient clinic during pregnancy. HCMV IgG and IgM tests were ordered routinely for pregnant women as part of TORCH surveillance. The age of pregnant women ranged from 17–45 years with the majority in their twenties and thirties. The TORCH test was ordered for children mainly if they presented with jaundice and/or prolonged fever of unknown origin. The age of children was between one month and 14 years. In case of newborns, HCMV IgG and IgM tests were ordered either as part of TORCH surveillance if any clinical manifestation suggestive of congenital infection was observed, or because they were offspring of HCMV IgM-positive mothers, even when signs of congenital infection were absent. Newborns were between one and 21 days old and were mainly tested using their mother’s name as identification as most of them had not yet been determined.

### Ethical approval

Patients’ data and results were made available by the director of the MICH Central Laboratory and approved by the MICH Ethics Committee. Reviewing medical files was approved by the MICH Ethics Committee.

### HCMV IgG and IgM tests

IgG and IgM were tested from serum samples using AxSYM kits (CMV IgG; 34-1685/R7 and IgM; 34-3532/R6) on Abbott AxSYM machine according to the manufacturer instructions. Tests were performed at the MICH Central Laboratory. The laboratory participates regularly in internationally recognized external quality control (Labquality, Helsinki, Finland) for HCMV serology three times annually.

### Avidity test

HCMV IgG avidity assay was performed on IgM-positive serum samples using Anti-CMV ELISA (EUROIMMUN Medizinische Labordiagnostika AG, Lübeck, Germany). HCMV IgG avidity assay had been introduced into the test list of the MICH Central Laboratory in January, 2012. The laboratory participates in internationally recognized external quality control (Labquality, Helsinki, Finland) for HCMV IgG avidity once annually.

### Real time PCR

Real time PCR for HCMV was performed at the Virology Laboratory at Al-Quds University. DNA was isolated from urine or whole blood, serum or urine samples using the QIAamp DNA Blood Mini Kit (cat#51304, Qiagen, Hilden, Germany). Real time PCR for viral load detection was performed using an ABI Real Time PCR 7500 (Applied Biosystems, USA). The reaction included TaqMan universal master mix (Applied Biosystems), CMV –gB-F: 5′-TGG GCG AGG ACA ACG AAA TC-3′, CMV –gB-R: 5′-TGA GGC TGG GAA GCT GAC AT-3′ as primer and 5′(FAM) -TGG GCA ACC ACC GCA CTG AGG – (BHQ) 3′ as probe (modified, [35]), respectively. All positive controls, negative controls and samples were tested in duplicate. A validated viral DNA (CMV_AD169_, quantitated DNA PCR control, cat# 08-925-000, Abionline, MD, USA) was used as a standard in all experiments. The standard was serially diluted in ultra pure water from 10^6^ down to 10^1^ copies/μl and dilutions were freshly prepared with each run.

### Data analysis

The results were collected from an Access data base program designed by the director of MICH Central Laboratory and copied into Excel spread sheets for analysis. Clinical presentations, data for HCMV viral load and HCMV avidity were analyzed separately.

## Results

In total, 1620 HCMV IgG and 1620 HCMV IgM tests performed for pregnant women, children, and newborns during the period from 2006–2012 were analyzed. In 1480 patients (91.4%) both IgG and IgM were tested simultaneously. In the remaining 8.6% of the patients, either IgG or IgM was tested. The following analysis was based only on those cases for which both tests were obtained simultaneously.

### Prevalence of HCMV IgG and IgM

**Figure 1:**
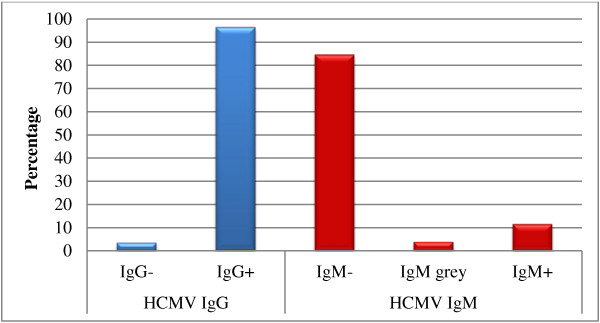
**Seroprevalence of IgG and IgM in Palestinian pregnant women.** The percentages of negative, positive and grey zone (in case of IgM) were calculated using the total number of women tested (556 cases).

**Figure 2:**
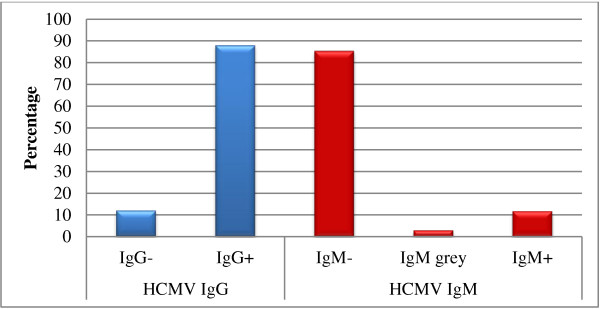
**Seroprevalence of IgG and IgM in hospitalized Palestinian children.** The percentages of negative, positive and grey zone (in case of IgM) were calculated using the total number of children tested (924 cases).

**Figure 3:**
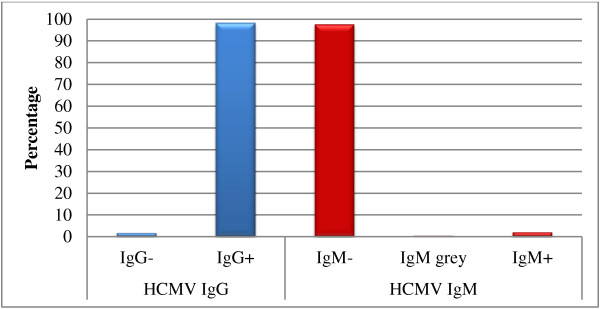
**Seroprevalence of IgG and IgM in hospitalized Palestinian newborns.** The percentages of negative, positive and grey zone (in case of IgM) were calculated using the total number of newborn tested (249 cases).

556 (37.6%) of analyzed records were those of pregnant women in their first trimester, 924 (62.4%) of children, 249 cases (26.9%) of which were newborns (Table 1). HCMV IgG was positive in 96.6% of women (Figure 1, Table 1) and in 88% of children respectively (Figure 2, Table 1). Further evaluation of the children showed that girls and boys tested equally positive for IgG. In newborns, IgG seropositivity was as high as 98.4% (Figure 3, Table 1).

**Table 1:** Status of HCMV serological markers IgG and IgM in the study population

**Study population**	**Total number**	**IgG + n (%)**	**IgG-n (%)**	**IgM +n (%)**	**IgM grey n (%)**	**IgM-n (%)**
**Pregnant women**	**556**	537 (96.6)	19 (3.4)	64 (11.5)	21 (3.8)	471 (84.7)
**Children (total)**	**924**	813 (88)	111 (12)	108 (11.7)	27 (2.9)	789 (85.4)
**Newborns**	**249**	245 (98.4)	4 (1.6)	5 (2)	1 (0.4)	243 (97.6)

Pregnant women, children and Newborns are indicated; number of cases (n) subjected to HCMV IgG and IgM testing is given, while the percentages are in parenthesis.

Regarding the IgM status of pregnant women, 84.7% were negative, 11.5% were positive and 3.8% were in the grey zone (Figure 1, Table 1). Five of the 63 IgM-positive women and two of the 21 grey zone IgM women became IgM negative when tested one month later. IgG and IgM were negative in 18 pregnant women (3.2%), while only one woman (0.2%) out of the total of 556 was negative for IgG and positive for IgM (Table 2).

**Table 2:** Verified HCMV IgG/IgM status in the study population

**Population**	**Pregnant Women**		**Children (total)**		**Newborns only**	
**Immunoglobulin**	**IgG+**	**IgG-**	**IgG+**	**IgG-**	**IgG+**	**IgG-**
	**n (%)**	**n (%)**	**n (%)**	**n (%)**	**n (%)**	**n (%)**
**IgM+**	63 (11.3)	1 (0.2)	104 (11.3)	4 (0.4)	5 (2)	0 (0)
**IgM-**	453 (81.5)	18 (3.2)	685 (74.1)	104 (11.3)	239 (96)	4 (1.6)
**IgM grey**	21 (3.8)	0 (0)	24 (2.6)	3 (0.3)	1 (0.4)	0 (0)

Pregnant women (556), children (924) including newborns (249) are indicated; number of cases (n) subjected to HCMV IgG and IgM testing is given, while the percentages are in parenthesis.

Among the children, 11.7% were IgM positive, 85.4% were negative and 2.9% had IgM values within the grey zone (Figure 2, Table 1). IgM positive status was 6% higher in boys than in girls. While 11.3% of children were IgG and IgM negative, another 11.3% were positive for both IgG and IgM. Seven children were found to be negative for IgG and were either positive or in the grey zone for IgM, while 24 were positive for IgG with grey zone IgM values (Table 2). Only 2% of the newborns tested were IgM positive and 0.4% fell into the grey zone IgM status (Figure 3, Table 1, Table 2).

### HCMV avidity

Sera of 40 HCMV IgM-positive women (62.5% of total IgM-positive women) were tested for HCMV IgG avidity. Twenty-six of these serum samples had been archived since 2010; the remaining 14 sample belonged to women who had tested positive for HCMV IgM in 2012. Thirty-nine women (97.5%) were positive for both IgG and IgM, while only one woman was IgG negative and IgM positive, for whom IgG avidity could not be tested. Another woman presented with 44% HCMV IgG avidity, which is in the grey zone according to the manufacturer of the kit. All the other 38 women (95%) presented with high HCMV IgG avidity (>60%).

### Detection of HCMV DNA

The urine of 10 (62.5%) out of 16 newborns (13 were born to IgM-positive mothers) tested positive for HCMV DNA. Viral load for two positive newborns was not tested at the Virology Research Laboratory. The urine of 15 (83.3%) out of 18 children (all IgM positive) and 3 out of 4 children (IgM grey zone) tested positive for HCMV DNA.

HCMV viral load was assayed in the whole blood of another 17 children between the ages of two months and 14 years. Eleven children were HCMV DNA viremic, six were HCMV IgM positive, while five were HCMV IgM negative.

Sixteen of the women tested for HCMV avidity were also tested for HCMV DNA in their sera. While three women tested negative for HCMV DNA, 13 were viremic (including the one IgG negative and IgM-positive case).

### Clinical observations

Medical charts of all 103 HCMV IgM-positive children, aged 23 days to 14 years, were reviewed and clinical presentations suggestive of HCMV infection were recorded. The major clinical presentations were hepatosplenomegaly or hepatomegaly (60%), followed by jaundice (30%). Prolonged fever or fever of unknown origin was recorded in 10% of the cases as one of the major clinical signs, while acute liver failure or fulminant liver failure presented in 8.7% of the cases. An ophthalmologic exam was ordered for 33 (32%) children, eight (24.2%) of whom were suffering from different eye findings, including chorioretinal spots, bilateral mild cataract, bilateral optic atrophy and bilateral choroidal coloboma. Hearing was impaired in one case. Nevertheless, out of all 103 children cases, only a single case, a two month-old child, suffering from progressive intrahepatic cholestasis was treated with Gancyclovir.

Medical registers of all 249 newborns tested for HCMV serology were reviewed. Sixteen newborns were subjected to HCMV viral load testing in urine; thirteen were born to mothers with recurrent HCMV infection. The remaining three newborns were admitted to MICH following birth (aged 1, 10 and 19 days) and tested for HCMV because of symptomatic congenital infection. The only cases treated with Gancyclovir, because of symptomatic congenital HCMV infection, along with viral shedding in urine, were one newborn born at MICH to a mother with recurrent HCMV infection and the three newborns admitted following birth. The first case suffered from HCMV chorioretinitis, the one-day-old newborn presented with microcephaly and intracerebral calcification, the 10-day-old infant (HCMV IgM positive) suffered from hepatosplenomegaly and neonatal jaundice, while the 19-day-old infant (HCMV IgM positive) presented with cholestatic jaundice and neurological manifestation. Interestingly, two of the mothers, whose newborns were admitted, tested positive for HCMV IgM on the admission of their infants (1-day and 10-day-olds). Finally, all infants, born at MICH to HCMV IgM-positive mothers were discharged with recommendations to follow up with ophthalmic test and auditory brain stem evoked response (ABER) hearing test, even when HCMV DNA in their urine was negative and congenital symptoms were absent.

## Discussion

The seroprevalence of HCMV IgG among pregnant Palestinian women and children as presented here is comparable to regional studies from Egypt [36], Qatar [30], Tunisia [37], Bahrain [38], Iraq [39], Iran [4],Turkey [28], [29]) and ranks among countries with high seroprevalence, comparable to Brazil, Cuba, Japan and Taiwan [24-27,40,41]. Lower regional seroprevalence is reported from Sudan at a rate of 72% among pregnant women [42] and Syria at a rate of 74.5% among college female students [43]. The seroprevalence among women in Israel varies based on ethnical groups. It has been shown to be as high as 80% in women of Afro-Asian origin and 65% in those of European-American origin [32]. The percentages are in fact 15% higher in both ethnic groups of women living in a Kibbutz environment [32]. The high level of IgG among newborns presented in this study is in accordance with earlier studies from regions of high maternal seroprevalence [27,36], normally due to placental transfer of maternal antibodies [44]. Taking into consideration the seroprevalence in children described here, we propose that the number of HCMV IgG-positive newborns would decline during the first months/years of childhood, to rise again in later childhood. Indeed, this has been observed in a recent study on HCMV IgG kinetics in Chinese newborns, where maternal seroprevalence is high [45].

The percentage of active infection (positive HCMV IgM) among pregnant Palestinian women is 3–5 times higher than that described in regional studies from Iran [4], Qatar [30], Sudan [42] and Turkey [28,29] or even international studies from Belgium, Brazil, Cuba, Finland and Taiwan [19,24,27,40,46]. A similar rate of active infection has been reported from Poland [47] and in one study from India [48]. The highly accurate AxSYM IgM test at MICH is a contributing technical factor for the high rate of positivity for IgM, as previously described [49]. Interestingly, only one woman (2.5%) out of the 40 IgM-positive Palestinian women tested was IgG negative, indicating a primary infection, while 38 (95%) presented with high HCMV IgG avidity. These data illuminate that recurrent infection among pregnant Palestinian women is the leading cause for this high level of active infection. Molecular biology studies are recommended to determine whether the recurrent infection is caused by a reactivation of a latent virus or reinfection with a new HCMV strain.

The percentage of positive HCMV IgM among hospitalized Palestinian children at MICH is also higher than percentages reported in a control group from Iraq [39] and Qatar [30]. Here, the high sensitivity of AxSYM IgM test is also a contributing technical factor for the high rate of positivity for IgM [49]. However, as mentioned in the methods section, MICH is the referral hospital in Palestine, so that children admitted to the pediatric ward are predominantly those with intractable medical conditions. Therefore, this IgM positive percentage and the high rate of HCMV viral load in urine and blood cannot be generalized to Palestinian children as an overall population. On the other hand, we propose that the medical conditions of at least a portion of these hospitalized children who tested positive for HCMV viremia or viral shedding in urine, as previously suggested, could be attributed to sequelae of undetected HCMV congenital infection [50,51].

Since vertical transmission of HCMV is 15-20% higher in primary infected women compared to women with recurrent infection [9,11,12,52], Palestinian infants may be considered at lower risk of perinatal HCMV infection, as most pregnant Palestinian women appear to suffer from recurrent infection. However, data from a recent study from in Israel demonstrates that the percentage of vertical transmission in women with recurrent infection is 19.6%, which is much higher than earlier estimations of 2.2% [53]. A large-scale study performed in Brazil has documented that the frequency of congenital HCMV disease in populations with high seroprevalence is higher than those with low seroprevalence [54]. In fact, a very recent review concludes that maternal HCMV seropositivity is a risk factor for congenital HCMV infection and HCMV-related hearing loss in children [55]. Taking these studies into consideration, the incidence of congenital HCMV infection in Palestine is probably underestimated due to the lack of large-scale studies. During this study period, four (1.6%) HCMV congenital infections out of 249 tested newborns were symptomatic, and thus were treated with Gancyclovir. This percentage does not reflect a true incidence rate of symptomatic congenital HCMV infection among Palestinian newborns, as not all women tested at MICH gave birth at the facility and three of these infants were admitted to MICH following birth. Viral shedding in urine was detected in another six babies, born to HCMV IgM-positive mothers, which may be associated with high risk of hearing loss in later childhood [56-58]. However, we have no records regarding the follow-up examinations, as most cases elect to consult physicians outside the MICH clinical facilities after discharge.

We have no records regarding the socioeconomic status (SES) of the women and children, whose results are presented here. Reports from various countries have shown that SES is proportional to the high rate of HCMV infection [9,13,21,33,46,59,60]. We have no evidence for SES playing such a critical role in the high rate of HCMV seroprevalence in Palestine. Therefore we propose rather that Palestinian social habits and living conditions may be the driving factor contributing to the high incidence of seroprevalence of HCMV.

## Conclusions

Screening pregnant women for HCMV IgG and IgM is not performed routinely in Palestine; MICH policy is thereby an exception. The data provided here and that of other studies strongly encourage routine testing for HCMV IgG and IgM among pregnant women in Palestine. Finally, follow-up tests with offspring to HCMV IgM-positive mothers are essential, in order to detect the consequences of congenital infection and to allow treatment to occur as early as possible.

## Competing interests

The authors declare that they have no competing interests.

## Authors’ contribution

TN, AQ, EA, FD and TN (Nusseibeh) performed the serology testing and samples’ archiving for DNA analysis. TN and AQ established and performed the Avidity testing. TN, AQ, EA and AA reviewed the patients’ files for clinical data. AA summarized the clinical findings and helped with literature review. NS and LQ performed the viral load testing. HK, MA and AA reviewed the newborns’ registers; HK reviewed the clinical observation summary presented here and approved it. TN and MA analyzed and verified the HCMV serology data. SB designed the program for data analysis, retrieved all serology tests and patients data available at the laboratory, verified and provided them for analysis. MA designed the study, summarized the data and wrote the paper. All authors read and approved the final manuscript.

## Pre-publication history

The pre-publication history for this paper can be accessed here:

http://www.biomedcentral.com/1471-2334/13/528/prepub
